# Maintenance of Mental Health: The Role of Physical Activity Among Young Adults

**DOI:** 10.3390/healthcare13222901

**Published:** 2025-11-13

**Authors:** Luca Szabó, Bettina F. Piko

**Affiliations:** 1 Doctoral School of Education, University of Szeged, 6722 Szeged, Hungary; szabo.luca@med.u-szeged.hu; 2 Department of Behavioral Sciences, University of Szeged, 6722 Szeged, Hungary

**Keywords:** physical activity, mental health, resilience, burnout, perceived stress, well-being, university students, cross-sectional study, convenience sampling

## Abstract

**Background:** Young adults, particularly university students, are at increased risk for psychological distress and burnout. Regular physical activity is widely recognized as a protective factor for mental health. This study aimed to compare physically active and inactive college students in terms of perceived stress, well-being, burnout, and resilience. **Methods:** A cross-sectional online survey was conducted among Hungarian university students (N = 264; 24.6% male; mean age = 24.21 years). Participants completed validated questionnaires assessing physical activity habits, perceived stress, resilience, and academic burnout. **Results:** Results showed that students engaging in regular physical activity reported significantly lower levels of perceived stress (Cohen’s d = 0.288) and burnout (Cohen’s d = 0.277), and higher resilience (Cohen’s d = 0.258) and well-being (Cohen’s d = 0.322) compared to their inactive peers. Correlation analyses confirmed strong associations between lower stress, reduced burnout, and greater resilience and well-being. Cluster analysis revealed two distinct psychological profiles: one characterized by higher mental hazards (stress and burnout) and the other by higher mental assets (resilience and well-being). Physical activity was strongly associated with membership in the mentally resilient cluster. **Conclusions:** These findings underscore the mental benefits of remaining physically active and highlight the importance of physical activity within university settings as a key strategy to enhance resilience, reduce academic burnout, and support the maintenance of mental health among young adults. However, the cross-sectional design, reliance on self-report measures, and convenience sampling limit causal interpretation and generalizability.

## 1. Introduction

The positive effects of sports on health have long been recognized. Numerous scientific studies have confirmed that regular physical activity plays a significant role both in maintaining health and in preventing diseases [1]. Within the young adult population, university students represent a group exposed to higher levels of stress, and student burnout is also common among them [2]. Entering higher education comes with a number of challenges that compound the developmental crises typical of young adulthood. Although student life largely results from individual choice, it can lead to experiencing various accidental crises: academic failures that undermine self-esteem, student burnout, or existential hazards [3]. It is a fundamental responsibility of higher education institutions to actively support the development and promotion of health-conscious lifestyles among their students by providing a motivating and supportive environment. In Hungary, physical education is mandatory and free of charge at several universities during four semesters [4]. As people age, the frequency of regular physical activity and sports participation generally tends to decline, making the university-age population the stage of life where significant improvements in this area can still be achieved. A study found that a significant proportion of university students in Hungary do engage in sports, even if not regularly; however, regular physical activity is often hindered by lack of time, fatigue, the desire for comfort, absence of a suitable social environment, and financial constraints [5]. The irregular schedule of university life, excessive workload, and lack of genuine community also contribute to students giving up their sports activities at the beginning of their studies [6]. Potential confounding factors, such as socioeconomic status, academic workload, and access to sports facilities, may also influence students’ participation in physical activity and should be considered when interpreting the results [5]. Recent international evidence confirms that these barriers are not unique to Hungary. Systematic reviews have consistently identified lack of time, academic pressure, and low motivation as the most frequent obstacles to physical activity among university students. Approximately half of students cite time constraints and study-related workload as the main reasons for physical inactivity [7,8]. These findings highlight the global relevance of this issue and underscore the importance of addressing both structural and motivational barriers when promoting regular exercise in higher education.

Perceived stress is a very common experience among college and university students, given the large amount of responsibilities and daily pressures they face [9]. Research has demonstrated a strong negative relationship between physical activity and perceived stress, with individuals who regularly exercise reporting markedly lower levels of stress [10]. In particular, physical activity was found to reduce evening stress. On stressful days, higher levels of physical activity were associated with increased positive affect and decreased negative affect [11]. Von Haaren et al. [12] implemented an aerobic exercise intervention with sedentary students and found that participants in the intervention group exhibited reduced autonomic nervous system reactivity and lower emotional responses to stressors compared to those in the control group. Flueckiger et al. [13] studied students over an academic year and found that on days when individuals were more physically active, stress had less impact on their mood. De Camargo et al. [14] investigated the effects of home-based physical activity on stress levels. They found that a low weekly frequency of physical activity significantly increased the likelihood of experiencing high levels of stress. In men, engaging in physical activity 4–5 times per week was sufficient to reduce stress, whereas in women, even higher frequencies of activity showed no significant association with stress levels, reflecting a greater susceptibility to stress in the female population.

There is a close relationship between academic stress and burnout among students in the higher education setting [3]. The concept of burnout was first introduced by Freudenberger [15], who described it as a psychological condition arising from prolonged work-related stress, characterized by exhaustion, loss of motivation, and the predominance of negative emotions. Later, Maslach and Jackson [16] refined this definition, identifying three core dimensions of burnout: exhaustion, cynicism, and reduced efficacy. In this framework, exhaustion refers to a persistent sense of fatigue and an inability to effectively perform study- or work-related tasks; cynicism (or emotional detachment) reflects an indifferent or negative attitude toward the meaning and value of one’s studies or professional activities; and reduced academic efficacy (inefficacy) involves feelings of incompetence and a diminished sense of accomplishment [17]. In academic contexts, as outlined by Schaufeli et al. these dimensions manifest specifically among students: exhaustion refers to students’ sense of being overwhelmed by study demands and constant academic pressure; cynicism manifests as growing indifference or detachment toward one’s coursework and academic environment; and reduced academic efficacy appears as students feeling unable to meet academic expectations, perceiving themselves as less capable and achieving less in their studies [18]. Research indicates that there is a complex relationship between participation in sports and academic burnout, and that engaging in moderate physical activity can help reduce the occurrence of academic burnout by alleviating stress and enhancing students’ sense of self-efficacy [19]. A study found that higher levels of physical exercise were significantly associated with lower academic burnout among college students. Physical activity directly reduced burnout and also indirectly did so by enhancing self-efficacy and resilience, highlighting exercise as an effective intervention target for preventing academic burnout [20].

Physical activity is a potential focus of intervention for enhancing well-being [21]. According to the results of a meta-analysis by Buecker and colleagues, physical activity significantly enhances subjective well-being, particularly playing a role in the development of positive affect [22]. Incorporating light daily activities, such as walking, may help mitigate the negative effects of stressful situations and improve mental well-being [11]. Examining the relationship between physical activity and well-being, optimism may contribute to better health behavior and thus improve physical and mental health [23]. Zhang and Chen [24] investigated the relationship between happiness and physical activity and found a positive association between the two factors. Based on the study by Roman et al. [25], there is a bidirectional, mutually reinforcing “virtuous cycle” between mental well-being and physical activity, in which higher well-being promotes greater physical activity, which in turn further enhances mental well-being.

Another mental asset, resilience, refers to an individual’s dynamic adaptive capacity that enables effective coping with stress, trauma, and other challenging life events across various contexts. It is not an innate trait but rather a continuously developable adaptive skill that can be strengthened through targeted interventions and life experiences [26,27]. Physical activity provides an appropriate environment for developing an individual’s psychological resilience [28]. Numerous studies have found a positive correlation between physical activity and psychological resilience [29,30]. According to Li et al. [31], physical activity significantly increased students’ resilience, which in turn contributed to a reduction in negative emotions.

The aim of this study has been to compare two groups of college students: those who engage in physical activity regularly with their non-active peers. Specifically, we investigate how these groups differ across key dimensions of mental health, including perceived stress, well-being, burnout, and resilience. This study contributes to the existing literature by simultaneously examining multiple mental health indicators (perceived stress, burnout, resilience, and well-being) in a Hungarian university population using a cluster-analytical approach. Since there is a considerable decline in university students’ physical activity compared to younger age groups, we have decided to test differences in mental health between those who are still physically active and those who are not. We hypothesize that the regular physical activity status is associated with better mental health outcomes, characterized by lower perceived stress and burnout, and higher levels of well-being and resilience.

## 2. Materials and Methods

### 2.1. Study Sample and Design

This study employed a cross-sectional, online survey design. A total of 264 university students participated, of whom 24.6% were male, aged 18–35 years (mean age, M = 24.21 years). Power calculations indicated that the sample size of 264 provides sufficient statistical power to detect small to medium effects. Due to convenience sampling, the total population is unknown. During the time period of data collection, altogether, 264 voluntary participants filled in the online questionnaire. Computing the minimum sample size to meet the desired statistical constraints, we applied the sample size calculator, with a result that our sample size proved acceptable with a 95% CI and 6% margin of error. Participants were recruited via Facebook groups and university-targeted social media platforms using convenience sampling. Completion of the questionnaire took approximately 15–20 min. Participation was voluntary and anonymous, and no personally identifiable data were collected. Ethical approval was obtained from the Doctoral School of Education, University of Szeged (approval number: 25/2024). Data collection took place in a time period between December 2024 and September 2025.

### 2.2. Questionnaires

The online questionnaire collected information on demographics, sport participation, and key psychological constructs, including perceived stress, resilience, and academic burnout.

The first section of the questionnaire focused on participants’ physical activity habits. Respondents were asked whether they engage in regular exercise, the duration of their involvement, and the type of sport practiced. Additionally, the questionnaire assessed the frequency and weekly duration of physical activity, average time spent per session, whether they exercise multiple times per day, and whether they participate in the sport at a competitive level.

Perceived stress was assessed using the 14-item Perceived Stress Scale (PSS) [32], adapted to Hungarian by Stauder and Konkolÿ Thege [33], with responses on a 5-point Likert scale (1 = never, 5 = very often). The scale measured participants’ subjective stress over the past month, including feelings of unpredictability, lack of control, and overload. This measurement demonstrated good internal consistency in the present sample (Cronbach’s α = 0.86).

Academic burnout was assessed with the School Burnout Inventory [34], Hungarian modified version by Jagodics et al. [35], comprising three subscales for exhaustion, cynicism, and feelings of inadequacy. Participants rated the 9 items on a 5-point Likert scale (1 = not at all true for me, 5 = completely true for me), and internal consistency was good (Cronbach’s α = 0.88).

The WHO Well-Being Index was used to measure general well-being [36], using the Hungarian validated version [37]. The 5-item scale included statements about the respondents’ feelings during the past two weeks. A 4-point Likert scale was available for the responses, ranging from 0 (at no time) to 3 (all of the time). Higher scores indicated a greater level of well-being. The reliability coefficient (Cronbach’s alpha) was α = 0.84 with this sample.

Finally, resilience was measured using the 10-item Connor–Davidson Resilience Scale (CD-RISC) [38], Hungarian adaptation by Járai et al. [39]. Items were rated on a 5-point Likert scale (0 = not true at all, 5 = almost always true) and total scores ranged from 0 to 50, with higher scores indicating greater resilience. The scale showed strong internal reliability in the current sample (Cronbach’s α = 0.86).

### 2.3. Statistical Analysis

Data were analyzed using IBM^®^ SPSS^®^ Statistics 26.0. No missing data were present in the dataset, so no specific handling was required. First, frequencies for sporting habits were displayed. Then, descriptive statistics for the psychological scales were obtained, while *t*-tests were calculated to detect differences in these scales by gender and sporting status. Pearson correlations tested the bivariate relationship between the study scales. Subsequently, k-means cluster analysis was performed based on the associations between variables, and participant profiles were determined. Finally, we examined cluster membership by group differences in sporting status.

## 3. Results

### 3.1. Demographic and Sporting Characteristics

The study included 264 university students (24.6% male) ranging in age from 18 to 35 years (M = 24.21). Among the participants, 64.02% reported engaging in regular or occasional physical activity, whereas 35.98% were classified as inactive. The sample’s sporting characteristics are summarized in Table 1.

### 3.2. Descriptive Statistics

Table 2 presents the descriptive statistics for the study scales (N = 264). The results indicate that students reported a perceived stress score of M = 40.58 (SD = 8.53), reflecting notable psychological strain related to daily challenges. The mean score for student burnout was M = 23.07 (SD = 8.93), while the resilience score averaged M = 28.19 (SD = 6.74). The mean level of well-being was M = 9.91 (SD = 3.39). Skewness and kurtosis values for all variables were within the acceptable range (±1), indicating that the data were approximately normally distributed and suitable for further statistical analyses. Therefore, a separate normality test (e.g., Shapiro–Wilk) was not conducted.

### 3.3. Differences by Gender and Exercise Status

Based on the results of Table 3, women reported higher perceived stress levels compared to men [t(264) = −2.079, *p* = 0.039], whereas there were no significant gender differences in student burnout, resilience, or well-being (*p* > 0.05). When examining sporting status, students who engaged in regular physical activity exhibited lower perceived stress [t(264) = 2.215, *p* = 0.028] and burnout [t(264) = 2.157, *p* = 0.032] scores, as well as higher resilience [t(264) = −1.997, *p* = 0.047] and well-being [t(264) = −2.549, *p* = 0.011] levels compared to their non-active peers. Table 3 presents the descriptive statistics of the studied variables and the differences according to gender and sporting status.

### 3.4. Differences by Other Sporting Characteristics

As shown in Table 4, students with longer sporting experience (≥10 years) reported significantly lower levels of student burnout (*t*(167) = 2.21, *p* = 0.028, *d* = 0.30), higher resilience (*t*(167) = –2.02, *p* = 0.044, *d* = 0.28), and well-being (*t*(167) = –2.08, *p* = 0.039, *d* = 0.28), compared to those with less than 10 years of sporting experience. The difference in perceived stress approached significance (*t*(167) = 1.82, *p* = 0.070, *d* = 0.24), suggesting a possible trend toward lower stress among long-term exercisers. Regarding the level of sporting activity, competitive athletes showed significantly lower burnout than hobby-level participants (*t*(167) = 1.31, *p* = 0.032, *d* = 0.33), whereas no significant differences were found for perceived stress, resilience, or well-being (*p* > 0.05). Finally, exercising more than once a day did not result in significant differences across any of the psychological indicators (all *p* > 0.30). We also tested the role of weekly and occasional sporting time using ANOVA, but none of these variables showed significant differences in the scales.

### 3.5. Bivariate Association Between the Study Scales

Based on the zero-order correlations, sporting status showed a significant negative relationship with perceived stress (*r* = −0.136, *p* < 0.05) and student burnout (*r* = −0.132, *p* < 0.05), while it was positively associated with resilience (*r* = 0.122, *p* < 0.05) and well-being (*r* = 0.156, *p* < 0.05). Perceived stress was strongly and positively related to student burnout (*r* = 0.494, *p* < 0.001) and strongly but negatively related to resilience (*r* = −0.586, *p* < 0.001) and well-being (*r* = −0.537, *p* < 0.001). Student burnout showed negative correlations with resilience (*r* = −0.326, *p* < 0.001) and well-being (*r* = −0.435, *p* < 0.001), whereas resilience was positively associated with well-being (*r* = 0.500, *p* < 0.001) (Table 5).

**Figure 1:**
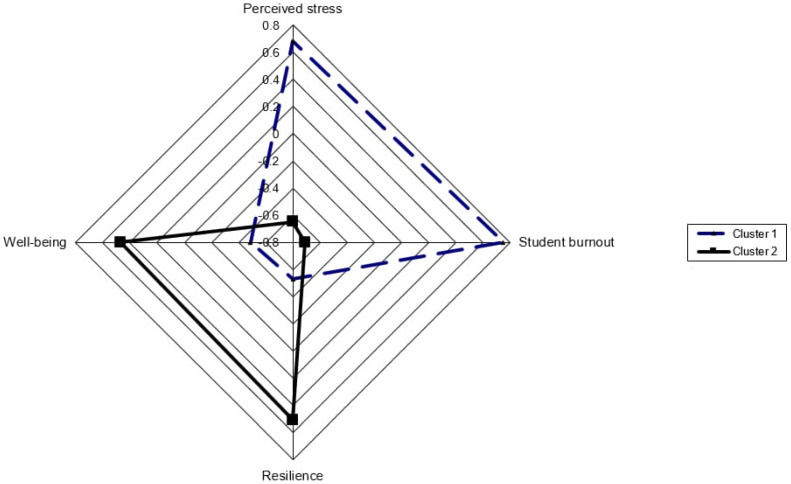
Cluster profiles based on z-scores.

In order to determine the participants’ profiles according to the mental assets and hazards, K-means clustering analysis was conducted. A priori, the Elbow method and dendrogram identified the point (k = 2) for the optimal number of clusters, based on the concept that adding more clusters only minimally decreased the total cluster variance. The total mean silhouette score was 0.50, indicating a moderate level of cluster cohesion and separation, suggesting only fair distinctiveness between clusters, which should be interpreted with caution. The means, standard deviations, z-scores, and F-values of the clusters are shown in Table 6, while cluster profiles based on z-scores are depicted in Figure 1.

Cluster 1 included 129 participants (48.86%) with higher levels of perceived stress and student burnout (i.e., mental hazards) and lower levels of resilience and psychological well-being (i.e., mental benefits). In contrast with these characteristics, Cluster 2 included 135 participants (51.14%) with higher levels of well-being and resilience, and lower levels of burnout and perceived stress (the opposite direction). Thus, we can describe Cluster 1 as characterized by higher mental hazards, including elevated perceived stress and student burnout, alongside lower mental assets such as resilience and psychological well-being, while Cluster 2 can be described as characterized by higher mental assets, with greater resilience and well-being, and lower perceived stress and burnout. Based on *t*-values and Cohen’s d values, the two clusters are well-separated along these variables (*p* < 0.001). The effect size is above 0.80 in each case, which can be classified as large. Results with ANOVA also justified a significant difference in each case (*p* < 0.001).

Finally, there was no association between the cluster belongingness and sex (χ^2^[1, n = 264] = 0.253, *p* = 0.616, Phi = 0.031). On the other hand, the relationship was significant with the sporting status (χ^2^[1, n = 264] = 7.366, *p* = 0.007, Phi = 0.167) and the length of sporting time (χ^2^[1, n = 264] = 5.459, *p* = 0.019, Phi = 0.144), Table 7.

## 4. Discussion

The present study examined the associations between physical activity, mental assets (resilience and well-being), and mental hazard (perceived stress and burnout) among university students. Consistent with our hypotheses, students who engaged in regular physical activity reported lower levels of perceived stress and burnout, as well as higher levels of resilience and well-being, compared to their inactive peers. These findings align with a substantial body of literature emphasizing the protective role of physical activity in psychological functioning and stress management [10,12,22].

Our results reinforce previous observations that regular exercise is associated with lower perceived stress by regulating physiological stress responses and enhancing positive affect [11]. The negative correlation between physically active status and burnout found in this study is also in line with earlier findings suggesting that exercise was associated with lower academic exhaustion and disengagement by promoting self-efficacy and emotional resilience [19,20]. Moreover, the positive association between resilience and well-being underscores the notion that resilience serves as a mediating mechanism through which physical activity supports mental health [29,31].

Gender differences in perceived stress were modest but significant, with female students reporting higher stress levels than males. This is consistent with prior research indicating that women may experience greater emotional reactivity to academic and social stressors [14]. Potential explanations for higher stress levels among female students include greater reliance on emotion-focused coping and the more frequent use of strategies such as self-distraction, emotional support, instrumental support, and venting [40]. These findings underscore the importance of considering gender-specific coping patterns when designing interventions, such as stress management programs or physical activity initiatives tailored to female students, to effectively support their mental well-being. However, no gender differences were found in burnout, resilience, or well-being. We should also take into account that recent Central European research has demonstrated significant long-term consequences of COVID-19 on both mental and physical health in young adults. In their sample of 18–30-year-olds, female participants reported notable post-COVID declines in concentration, memory, and physical fitness, with physical inactivity strongly associated with these impairments. These findings highlight that promoting regular physical activity may be particularly important for supporting cognitive performance and overall recovery among female university students in the post-pandemic era [41].

The present findings highlight that both the duration and level of sporting involvement are important factors in students’ mental health. Participants with a longer history of regular sport engagement showed lower burnout and higher resilience and well-being compared to those with shorter experience. This suggests that long-term participation in physical activity may have cumulative psychological benefits, enhancing stress-coping capacity and emotional stability over time. The tendency toward lower perceived stress among long-term exercisers also supports the notion that continuous engagement in sport helps protect against chronic stress and emotional exhaustion [12,31]. Students involved in competitive sports displayed slightly lower levels of burnout than those participating only at a recreational level. This pattern may reflect the added psychological resources associated with structured training environments, clear goal setting, and the social support provided within competitive sport contexts. Such findings are consistent with previous evidence that higher levels of engagement in sport contribute to stronger self-efficacy and resilience, both of which are crucial protective factors against stress and burnout [19,20]. In contrast, the frequency of exercising per day did not appear to influence psychological outcomes, suggesting that the quality, consistency, and personal meaning of physical activity might be more important than short-term intensity [22,25]. Taken together, these results emphasize that long-term and committed engagement in sport—regardless of competitive level—can serve as a sustainable resource for maintaining mental well-being among university students.

The cluster analysis identified two distinct psychological profiles among participants: one characterized by high mental hazards (elevated stress and burnout) and the other by high mental assets (strong resilience and well-being). Although the cluster analysis revealed two distinct profiles, the moderate silhouette score (0.50) indicates only fair separation between clusters, limiting generalizability. The strong relationship between cluster membership and physical activity status highlights the broader psychosocial benefits of exercise and underscores the potential of sports participation as a tool for promoting mental sustainability within university populations. These results support the conceptual model of a “virtuous cycle” between physical activity and mental health [25], wherein engagement in exercise enhances well-being, which in turn encourages further participation in health-promoting behaviors.

From a practical perspective, these findings suggest that universities should invest in programs and infrastructure that promote regular physical activity, such as accessible sports facilities, flexible physical education requirements, and community-based wellness initiatives. Encouraging even moderate levels of activity is associated with higher psychological resilience and overall mental sustainability, as physical activity may enhance students’ ability to cope with academic and social challenges, regulate stress responses, and maintain positive affect. These effects may operate through multiple mechanisms: physiologically by reducing cortisol levels, psychologically by increasing self-efficacy and resilience, and socially by strengthening social support networks [42]. Furthermore, by reducing symptoms of academic burnout—a major predictor of student dropout [3]—regular physical activity may indirectly contribute to higher academic persistence and retention rates within higher education institutions.

The strength of our paper is to draw attention to several indicators of mental health, including both mental assets and mental hazards. Our findings may trigger further research to explore associations of physical activity with other potential mental health indicators and their mechanism. In addition, we used scales that have been previously validated on Hungarian populations. Despite these strengths, several limitations must be acknowledged. As our study is a cross-sectional one, the findings demonstrate associations rather than causal relationships. Moreover, because participants were recruited through an online convenience sample, a potential self-selection bias cannot be ruled out, as students more interested in health-related topics may have been more likely to participate. The cross-sectional design precludes causal inference, and the use of self-reported measures may have introduced response bias. Physical activity was assessed using a brief self-report question rather than a validated questionnaire, which limits the precision and comparability of the measure. We used the prevalence of being physically active or not, i.e., a simplified measure of sporting status, and the relatively low sample size did not allow us to use more sophisticated measurements, e.g., sports types. Furthermore, the binary categorization of physical activity (active vs. inactive) oversimplifies this complex behavior, as it does not capture variations in activity type, intensity, duration, or enjoyment. Additional limitations include the low representation of male students in our sample (24.6%), which may affect the generalizability of the findings across genders. Cultural specificity to Hungary should also be considered, as both university policies and societal attitudes towards physical activity may differ from other contexts, potentially limiting the broader applicability of the results. These limitations may have influenced the interpretation of the findings.

## 5. Conclusions

In conclusion, the current study adds to the growing evidence that physical activity is a critical factor in maintaining mental health and well-being among young adults. Promoting regular exercise is associated with lower levels of psychological distress and academic burnout, as well as higher resilience and well-being—key components of maintaining favorable mental functioning. While offering a wide range of benefits from physical and mental health to academic success and social integration, engagement in sports activity tends to decline during the university years [5,6]. Universities should play a crucial role in student engagement in sports by providing structured programs and promoting both recreational and competitive opportunities [43]. Evidence from systematic reviews indicates that complex, multi-strategy interventions in university settings (e.g., combining health-promotion education, incentives, digital tools) are effective for increasing physical activity among students [44].

Future research should employ longitudinal or experimental designs, including randomized controlled trials, to clarify causal pathways and to examine potential mediators, such as self-efficacy or social support. Additionally, qualitative investigations could provide deeper insights into the motivational and contextual factors influencing students’ engagement in physical activity. It would also be valuable to examine risk populations, such as students seeking counseling or life-management support services, in order to assess whether promoting regular physical activity within these groups leads to measurable improvements in mental health outcomes over time through longitudinal research designs. Finally, further studies should be expanded to include digital lifestyle, sedentary behavior, or socioeconomic barriers (e.g., screen time, financial constraints, pandemic effects) that may affect students’ capacity for physical activity, also focusing on sex-specific associations.

## Figures and Tables

**Table 1:** Sporting characteristics of the sample (N = 264).

Variables (N = 264)	n	%
Sex		
Male	65	24.60
Female	199	75.42
Sporting status		
Inactive	95	35.98
Active	169	64.02
**Variables (N = 169)**	**n**	**%**
Length of sporting time (years)		
1 year or less	25	14.79
2–3 years	28	16.57
3–5 years	16	9.47
5–10 years	23	13.61
>10 years	77	45.56
Sporting on days/week		
1 day	59	34.91
2 days	74	43.79
3 days	31	18.34
4 days or more	5	2.96
Sporting time per occasion		
1–2 h	81	47.93
3–4 h	81	47.93
5 h or more	7	4.14
More than one occasion a day		
No	117	69.23
Yes	52	30.77
Level of sporting		
Hobby	148	87.57
Competitive	21	12.43
Type of sport		
Endurance	121	71.60
Tactical	19	11.24
Combat	12	7.10
Artistic	17	10.06

**Table 2:** Descriptive statistics for study variables (N = 264).

Variables	Range	Mean (SD)	Skewness	Kurtosis
Perceived stress	18–65	40.58 (8.53)	−0.103	−0.058
Student burnout	9–45	23.07 (8.93)	0.424	−0.718
Resilience	0–40	28.19 (6.74)	−0.734	0.552
Well-being	0–15	9.91 (3.39)	−0.309	–0.610

**Table 3:** Descriptive statistics by gender and sporting status (N = 264).

	Sex			Sporting Status		
	Male (n = 65)	Female (n = 199)		No (n = 95)	Yes (n = 169)	
Variables	Mean (SD)	Mean (SD)	*t*-Value Significance Cohen’s d	Mean (SD)	Mean (SD)	*t*-Value Significance Cohen’s d
Perceived stress	38.68 (8.42)	41.20 (8.50)	−2.079 *p* = 0.039 0.298	42.12 (7.98)	39.71 (8.73)	2.215 *p* = 0.028 0.288
Student burnout	23.22 (8.72)	23.03 (9.02)	0.149 *p* = 0.882 0.021	24.64 (8.74)	22.19 (8.94)	2.157 *p* = 0.032 0.277
Resilience	27.14 (6.97)	28.54 (6.64)	−1.456 *p* = 0.147 0.206	27.09 (6.51)	28.81 (6.81)	−1.997 *p* = 0.047 0.258
Well-being	9.66 (3.59)	9.99 (3.33)	−0.688 *p* = 0.492 0.096	9.21 (3.63)	10.31 (3.19)	−2.549 *p* = 0.011 0.322

Note. Student’s *t*-test.

**Table 4:** Descriptive statistics by other sporting variables I. (N = 264).

	Length of Sporting Time			Level of Sporting			More Than One Occasion a Day		
	<10 year (n = 92)	≥10 years (n = 77)		Hobby (n = 148)	Competitive (n = 21)		Yes (n = 52)	No (n = 117)	
Variables	Mean (SD)	Mean (SD)	*t*-Value Significance Cohen’s d	Mean (SD)	Mean (SD)	*t*-Value Significance Cohen’s d	Mean (SD)	Mean (SD)	*t*-Value Significance Cohen’s d
Perceived stress	41.19 (8.29)	39.09 (8.99)	1.822 *p* = 0.070 0.243	39.93 (8.79)	38.19 (8.36)	0.852 *p* = 0.396 0.203	40.73 (8.99)	39.26 (8.68)	1.013 *p* = 0.312 0.167
Student burnout	23.84 (8.94)	21.19 (8.68)	2.208 *p* = 0.028 0.301	22.53 (9.11)	19.81 (7.35)	1.307 *p* = 0.032 0.329	22.94 (10.39)	21.85 (8.23)	0.729 *p* = 0.467 0.116
Resilience	27.66 (6.80)	29.49 (6.46)	−2.023 *p* = 0.044 0.276	28.80 (6.70)	28.86 (7.71)	−0.033 *p* = 0.973 0.008	28.81 (7.32)	28.81 (6.60)	−0.004 *p* = 0.997 0.000
Well-being	9.64 (3.44)	10.58 (3.19)	−2.077 *p* = 0.039 0.283	10.31 (3.21)	10.29 (3.15)	0.034 *p* = 0.973 0.006	10.31 (3.36)	10.31 (3.13)	0.000 *p* = 1.000 0.000

Note. Student’s *t*-test.

**Table 5:** Zero-order correlations for the study variables (N = 264).

Variable (Scores)	2	3	4	5
1. Sporting status	−0.136 *	−0.132 *	0.122 *	0.156 *
2. Perceived stress	-	0.494 **	−0.586 **	−0.537 **
3. Student burnout	-	-	−0.326 ***	−0.435 **
4. Resilience	-	-	-	0.500 **
5. Well-being	-	-	-	-

Notes. *r* = correlation coefficients: * *p* < 0.05, ** *p* < 0.001, *** *p* < 0.001.

**Table 6:** Means, SD, z-scores, and *t*-test for psychological benefits and hazards (N = 264).

	Cluster 1 Mean (SD) z-Score	Cluster 2 Mean (SD) z-Score	*t*-Value	Cohen’s d
Perceived stress	46.40 (6.19) 0.68	35.01 (6.52) −0.65	14.524 *	1.792
Student burnout	29.75 (7.36) 0.75	16.69 (6.55) −0.71	17.427 *	1.875
Resilience	24.62 (6.53) −0.53	38.61 (5.00) 0.51	−9.833 *	2.406
Well-being	8.26 (3.37) −0.49	11.50 (2.56) 0.47	−8.821 *	1.027
N (percentage)	129 (48.86%)	135 (51.14%)		

Notes. * *p* < 0.001.

**Table 7:** Relationship of cluster belongingness with sex, sporting status, and the length of sporting time.

Variables	Cluster 1 n (%)	Cluster 2 n (%)	Chi-Square Tests
Sex			
Male	30 (46.2%)	35 (53.8%)	χ^2^ = 0.253 *p* = 0.615 Phi = 0.031
Female	99 (49.7%)	100 (50.3)	
Sporting status			
No	57 (60.0%)	38 (40.0%)	χ^2^ = 7.366 *p* = 0.007 Phi = 0.167
Yes	72 (42.6%)	97 (57.4%)	
Length of sporting time			
<10 years	100 (53.5%)	87 (46.5%)	χ^2^ = 5.459 *p* = 0.019 Phi = 0.144
≥10 years	29 (37.7%)	48 (62.3)	
		

## Data Availability

The data presented in this study are available in the Open Science Framework repository (OSF) at [https://osf.io/p6un7/overview?view_only=942d0e87599f4474b2ffab8a3528aae0, accessed on 19 October 2025].

## References

[1] Geidne S., Van Hoye A. (2021). Health promotion in sport, through sport, as an outcome of sport, or health-promoting sport—What is the difference?. Int. J. Environ. Res. Public Health.

[2] Olson N., Oberhoffer-Fritz R., Reiner B., Schulz T. (2021). Stress, student burnout and study engagement—A cross-sectional comparison of university students of different academic subjects. BMC Psychol..

[3] Zhang J., Meng J., Wen X. (2025). The relationship between stress and academic burnout in college students: Evidence from longitudinal data on indirect effects. Front. Psychol..

[4] Paár D., Kovács A., Stocker M., Hoffbauer M., Fazekas A., Betlehem J., Bergier B., Ács P. (2021). Comparative analysis of sport consumption habits in Hungary, Poland and Germany. BMC Public Health.

[5] Kosztin N., Balatoni I. (2021). Hungarian university students’ sporting habits: A literature review. Acta Med. Soc..

[6] Balatoni I., Varga Szepne H., Muller A., Kovacs S., Kosztin N., Csernoch L. (2019). Sporting habits of university students in Hungary. Balt. J. Health Phys. Act..

[7] Brown C.E.B., Richardson K., Halil-Pizzirani B., Atkins L., Yücel M., Segrave R.A. (2024). Key influences on university students’ physical activity: A systematic review using the Theoretical Domains Framework and the COM-B model of human behaviour. BMC Public Health..

[8] Silva R.M.F., Mendonça C.R., Azevedo V.D., Memon A.R., Noll P.R.E., Noll M. (2022). Barriers to high school and university students’ physical activity: A systematic review. PLoS ONE.

[9] Johnston S.A., Roskowski C., He Z., Kong L., Chen W. (2020). Effects of team sports on anxiety, depression, perceived stress, and sleep quality in college students. J. Am. Coll. Health.

[10] Schultchen D., Reichenberger J., Mittl T., Weh T.R., Smyth J.M., Blechert J., Pollatos O. (2019). Bidirectional relationship of stress and affect with physical activity and healthy eating. Br. J. Health Psychol..

[11] Hachenberger J., Teuber Z., Li Y.M., Abkai L., Wild E., Lemola S. (2023). Investigating associations between physical activity, stress experience, and affective wellbeing during an examination period using experience sampling and accelerometry. Sci. Rep..

[12] von Haaren B., Haertel S., Stumpp J., Hey S., Ebner-Priemer U. (2015). Reduced emotional stress reactivity to a real-life academic examination stressor in students participating in a 20-week aerobic exercise training: A randomised controlled trial using ambulatory assessment. Psychol. Sport Exerc..

[13] Flueckiger L., Lieb R., Meyer A.H., Witthauer C., Mata J. (2016). The importance of physical activity and sleep for affect on stressful days: Two intensive longitudinal studies. Emotion.

[14] de Camargo E.M., Piola T.S., Dos Santos L.P., de Borba E.F., de Campos W., da Silva S.G. (2021). Frequency of physical activity and stress levels among Brazilian adults during social distancing due to the coronavirus (COVID-19): Cross-sectional study. Sao Paulo Med. J..

[15] Freudenberger H.J. (1974). Staff Burn-Out. J. Soc. Issues.

[16] Maslach C., Jackson S.E. (1981). The Cost of Caring.

[17] Rosales-Ricardo Y., Ferreira J.P. (2022). Effects of physical exercise on Burnout syndrome in university students. MEDICC Rev..

[18] Schaufeli W.B., Martínez I.M., Marques-Pinto A., Salanova M., Bakker A.B. (2002). Burnout and engagement in university students: A cross-national study. J. Cross-Cult. Psychol..

[19] Fu W., Li Y., Liu Y., Li D., Wang G., Liu Y., Zhang T., Zheng Y. (2023). The influence of different physical exercise amounts on learning burnout in adolescents: The mediating effect of self-efficacy. Front. Psychol..

[20] Chen K., Liu F., Mou L., Zhao P., Guo L. (2022). How physical exercise impacts academic burnout in college students: The mediating effects of self-efficacy and resilience. Front. Psychol..

[21] Prendergast K.B., Schofield G.M., Mackay L.M. (2016). Associations between lifestyle behaviours and optimal wellbeing in a diverse sample of New Zealand adults. BMC Public Health.

[22] Buecker S., Simacek T., Ingwersen B., Terwiel S., Simonsmeier B.A. (2021). Physical activity and subjective well-being in healthy individuals: A meta-analytic review. Health Psychol. Rev..

[23] Boehm J.K., Chen Y., Koga H., Mathur M.B., Vie L.L., Kubzansky L.D. (2018). Is optimism associated with healthier cardiovascular-related behavior? Meta-analyses of three health behaviors. Circ. Res..

[24] Zhang Z., Chen W. (2019). A systematic review of the relationship between physical activity and happiness. J. Happiness Stud..

[25] Roman J.E.I., Ekholm O., Algren M.H., Koyanagi A., Stewart-Brown S., Hall E.E., Stubbs B., Koushede V., Thygesen L.C., Santini Z.I. (2023). Mental wellbeing and physical activity levels: A prospective cohort study. Ment. Health Phys. Act..

[26] Martín-Rodríguez A., Gostian-Ropotin L.A., Beltrán-Velasco A.I., Belando-Pedreño N., Simón J.A., López-Mora C., Navarro-Jiménez E., Tornero-Aguilera J.F., Clemente-Suárez V.J. (2024). Sporting mind: The interplay of physical activity and psychological health. Sports.

[27] Kim C., Yeom J., Jeong S., Chung J.B. (2023). Resilience and social change: Findings from research trends using association rule mining. Heliyon.

[28] Belcher B.R., Zink J., Azad A., Campbell C.E., Chakravartti S.P., Herting M.M. (2020). The roles of physical activity, exercise, and fitness in promoting resilience during adolescence: Effects on mental well-being and brain development. Biol. Psychiatry Cogn. Neurosci. Neuroimaging.

[29] Zhao Z., Zhao S., Wang Q., Zhang Y., Chen C. (2022). Effects of physical exercise on mobile phone addiction in college students: The chain mediation effect of psychological resilience and perceived stress. Int. J. Environ. Res. Public Health.

[30] Ho F.K.W., Louie L.H.T., Chow C.B., Wong W.H.S. (2015). Physical activity improves mental health through resilience in Hong Kong Chinese adolescents. BMC Pediatr..

[31] Li X., Yu H., Yang N. (2021). The mediating role of resilience in the effects of physical exercise on college students’ negative emotions during the COVID-19 epidemic. Sci. Rep..

[32] Cohen S., Kamarck T., Mermelstein R. (1983). A global measure of perceived stress. J. Health Soc. Behav..

[33] Stauder A., Konkolÿ Thege B. (2006). Characteristics of the Hungarian version of the Perceived Stress Scale. J. Ment. Health Psychosom..

[34] Salmela-Aro K., Kiuru N., Leskinen E., Nurmi J.-E. (2009). School burnout inventory (SBI). Eur. J. Psychol. Assess..

[35] Jagodics B., Kóródi K., Szabó É. (2021). Exploring the Student Burnout Scale using Hungarian sample. Hung. J. Psychol..

[36] Topp C.W., Østergaard S.D., Søndergaard S., Bech P. (2015). The WHO-5 Well-Being Index: A systematic review of the literature. Psychother. Psychosom..

[37] Susánszky É., Konkolÿ Thege B., Stauder A., Kopp M. (2006). Validation of The Short (5-item) version of The WHO Well-Being Scale based on a Hungarian Representative Health Survey (Hungarostudy 2002). J. Ment. Health Psychosom..

[38] Connor K.M., Davidson J.R.T. (2003). Development of a new resilience scale: The Connor–Davidson Resilience Scale (CD-RISC). Depress. Anxiety.

[39] Járai R., Vajda D., Hargitai R., Nagy L., Csókási K., Kiss E.C. (2015). Characteristics of the 10-item Connor–Davidson Resilience Scale. Appl. Psychol..

[40] Graves B.S., Hall M.E., Dias-Karch C., Haischer M.H., Apter C. (2021). Gender differences in perceived stress and coping among college students. PLoS ONE.

[41] Falbová D., Kovalčíková V., Beňuš R., Vorobeľová L. (2024). Long-term consequences of COVID-19 on mental and physical health in young adults. Cent. Eur. J. Public Health.

[42] Xu S., Liu Z., Tian S., Ma Z., Jia C., Sun G. (2021). Physical Activity and Resilience among College Students: The Mediating Effects of Basic Psychological Needs. Int. J. Environ. Res. Public Health.

[43] Diehl K., Fuchs A.K., Rathmann K., Hilger-Kolb J. (2018). Students’ motivation for sport activity and participation in university sports: A mixed-methods study. Biomed. Res. Int..

[44] García-Álvarez D., Faubel R. (2020). Strategies and measurement tools in physical activity promotion interventions in the university setting: A systematic review. Int. J. Environ. Res. Public Health.

